# Comparison of Biogenic Amorphous Silicas Found in Common Horsetail and Oat Husk With Synthetic Amorphous Silicas

**DOI:** 10.3389/fpubh.2022.909196

**Published:** 2022-06-22

**Authors:** Gottlieb Georg Lindner, Claus-Peter Drexel, Katrin Sälzer, Tobias B. Schuster, Nils Krueger

**Affiliations:** ^1^Evonik Operations GmbH, RD&I, Wesseling, Germany; ^2^Evonik Operations GmbH, Smart Materials, Hanau, Germany

**Keywords:** synthetic amorphous silica, biogenic silica, nanostructure, toxicity, common horsetail, oat husk, transmission electron microscopy (TEM), scanning electron microscopy (SEM)

## Abstract

The present study summarizes the current literature on the presence and the structure of biogenic amorphous silica (BAS) in nature. Based on this review, it is shown that BAS is ubiquitous in nature and exhibits a structure that cannot be differentiated from the structure of synthetic amorphous silica (SAS). The structural similarity of BAS and SAS is further supported by our investigations—in particular, specific surface area (BET) and electron microscope techniques—on oat husk and common horsetail. Many food products containing BAS are considered to be beneficial to health. In the context of the use of SAS in specific applications (e.g., food, feed, and cosmetics), this is of particular interest for discussions of the safety of these uses.

## Introduction

Synthetic amorphous silica (SAS) can be produced by either a vapor-phase hydrolysis process, yielding pyrogenic (fumed) silica, or by a wet process, yielding hydrated silica (precipitated silica, silica gel, or colloidal silica). During the manufacturing process of these SAS materials, the primary particles are strongly (covalently) bonded or fused to form aggregates (1). Physical attraction forces (van der Waals interaction and hydrogen bonding) lead to the formation of agglomerates in the micron-size range. Typically, isolated nanoparticles do not occur. In contrast, colloidal SAS dispersions may contain isolated primary particles in the nano-size range, which can be considered nano-objects. SAS powder is placed on the market as micron-sized aggregates and agglomerates with an internal structure in the nanoscale (ISO definition of nanostructured material).

Synthetic amorphous silicas can be regarded as nanomaterials (NM)^1^ and have been produced and marketed for many years. Fumed silica production began in 1944, and even in 1950, they had been described as “milli micron particles”; “milli micron” at this time was the equivalent of today's term “nano.” Interestingly, the current popular term “nano materials” (NMs) had been coined just a few years prior to the beginning of the new millennium. Since the great interest in NMs in the early 2000s, the word “nano” can be found in a wide variety of diverse scientific and technical fields. Following the establishment of the technical committee for nanotechnologies (ISO/TC 229) in 2005, international standardization evolved and finally, an NM is now defined by ISO/TS 80004-1 as “material with any external dimension in the nanoscale (1–100 nm) or having an internal structure or surface structure in the nanoscale.” This generic term includes nano-object and NM.

The new nomenclature of NMs led to the impression that these were “novel materials,” whereas it has been overlooked by the general public that some of these NMs have been deliberately produced and/or used either for many decades (e.g., SAS and carbon black), centuries (e.g., gold ruby glass), or even millennia (e.g., clay). In addition, there are many NMs naturally occurring in the environment originating from natural sources (e.g., volcanos and minerals *via* wear and abrasion) or from biological processes. We should also note the unintended environmental and occupational production of NMs, e.g., *via* combustion products (automobile engines and wildfires), welding, and other man-made or natural activities.

Biological processes, both in fauna and flora, not only synthesize organic NMs, such as liposomes but also inorganic NMs, such as iron oxides (e.g., magnetite in pigeons) or amorphous silica (e.g., in many kinds of grasses, such as rice, oat, wheat, or sugar cane; diatoms/algae, and sponges).

Helpfully, in the “ISO/TR 18401 Nanotechnologies—Plain language explanation of selected terms from the ISO/IEC 80004 series,” the nanostructured amorphous silica found in rice husk is named as an example of a naturally-occurring NM.

Synthetically manufactured NMs are strictly regulated in a number of industries. In this respect, extensive physicochemical, ecotoxicological, toxicological, safety, and epidemiological data have been collected for SAS, and no environmental or health risks have been identified provided SAS is produced and used under current occupational hygiene standards and recommendations for its safe use in various applications. It is therefore of interest to consider if these synthetic NMs can be differentiated from the ubiquitous, naturally occurring biogenic amorphous silica (BAS)^2^ NMs. In the present study, therefore, SASs (e.g., food additive E 551) are compared with their natural counterparts: BAS. It will be shown that the NMs of BAS are naturally present, especially in plants, and they can exhibit a similar nanostructure and surface area compared with SAS types used for food/cosmetics. In fact, due to their usual daily uptake, humans are evolutionarily adapted to the intake of BAS, whether it is in a particulate nanoform or dissolved. This is particularly relevant as the average human has about 1.5 g of silicon dioxide (SiO_2_) in their body (2). It is a vital chemical for all cells, and especially important for connecting tissues, such as the skin, bones, cartilage, tendons, and ligaments. It has been noted that the amount of silica in the body decreases with age (3–5).

Biogenic amorphous silica thus plays a vital role in the living environment and shows nanostructures that are very comparable with manufactured SAS. For oat husk (*Avena sativa)* and common horsetail (*Equisetum arvense*), their structures are even below 20 nm. This was shown for the latter from as early as 1991 (6). This present article reviews existing studies on the structure of BAS along with our study results. For the above-mentioned products, we have been able, for the first time, to compare the specific surface areas of SAS and BAS by Brunauer, Emmet, Teller (BET) and show that the results for BAS were as high as 150 m^2^/g.

## Materials and Methods

For our studies, we used samples of the common horsetail (*E. arvense*) supplied from Blanks GmbH & Co. KG (“Schachtelhalmkraut, geschnitten”), often used as a herbal tea. Oat husk (*A. sativa*) was obtained from Bio Bäckerei Spiegelhauer (“Haferspelzen, fein vermahlen”); usually this is often used for sprinkling proofing baskets in bread making.

### Sample Preparation for Both Common Horsetail and Oat Husk

To obtain an initial indication of the composition, a quantitative analysis of the content of SiO_2_ and other inorganic components in the horsetail and in the oat husks raw material was performed.

The samples were prepared according to the following procedure for the detailed investigation of the structure of the silicon dioxide present in the materials.

Schematic sample preparation steps for both raw BAS materials and naming of samples for later analysis.

- Starting material: raw material as received samples as received
- Digestion starts with about 30% hydrochloric acid at room temperature
- Multiple decanting/adding water = dilution (each cycle about 2–3 days) until HCl-concentration <0.05%
- Centrifugation at 20,000 rpm
- Drying at 105°C for 16 h
- Calcination (in small portions) at 540°C for 4 h—calcined without washing
- Thoroughly washing (employing ultrasonication and centrifuge)
- Drying at 105°C for 16 h—final sample

For comparison, samples of the raw materials were dried at 105°C (just dried; those have been calcined at 540°C for 4 h have been prepared (calcined raw materials), and washed with water (calcined raw materials/washed).

The common horsetail, as well as the oat husk, were digested with hydrochloric acid at room temperature for several days. Using successive dilution with deionized (DI) water, prolonged sedimentation, and decantation, the resulting greenish (common horsetail) to brown (oat husk) samples were then dried at 105°C overnight and subsequently calcined at 540°C for 4 h. This yielded a white powder which was thoroughly washed with DI water, centrifuged at 20,000 rpm, and finally dried at 105°C overnight.

In parallel, comparative samples were prepared for all steps, e.g., without digestion by hydrochloric acid, to ensure that any observed structures are not confounded by the sample preparation process.

### BET and Electron Microscopy

#### The Multi-Point BET Was Measured With an AUTOSORB® iQ Station

The scanning electron microscopy (SEM) imaging was done with a Jeol JSM-7600F/Oxford-EDX Aztec and the energy dispersive X-ray (EDX) analysis with Thermo Fisher Scientific Ultradry EDX. The transmission electron microscopy (TEM) analysis was performed with a Jeol 2010F. The quantitative analysis was done with an ICP-OES iCAP 6500 Duo (Thermo Fisher Scientific GmbH). Species preparation took place with a lithium-meta-borate melt.

## Results

### Quantitative Analysis of the Samples as Received

To determine the amounts of Na, Mg, K, Ca, P, Al, Fe, Ti, and SiO_2_ with ICP-OES, the samples were calcined at 540°C overnight and a lithium metaborate melt was used in the preparation of the measurements. The SiO_2_ content was calculated from the data for elemental Si. Values are given about the original biological sample as received (Table 1).

**Table 1:** Quantitative analysis of both horsetail and oat husk from samples “as received”.

		**Horsetail**	**Oat husk**
Loss on drying (105 °C over night	% Mass	8.4	6.8
Redidue on ignition 540 °C over night	% Mass	17.7	3.1
Na	μg/g	780	79
Mg	μg/g	53,00	330
K	μg/g	2.9	0.43
Ca	μg/g	1.7	0.04
P	μg/g	2,900	260
Al	μg/g	2,100	<10
Fe	μg/g	1,600	11
Ti	μg/g	140	<5
SiO_2_	% mass	6.8	2.2

### Results With Common Horsetail

#### Light Microscopy

**Figure 1:**
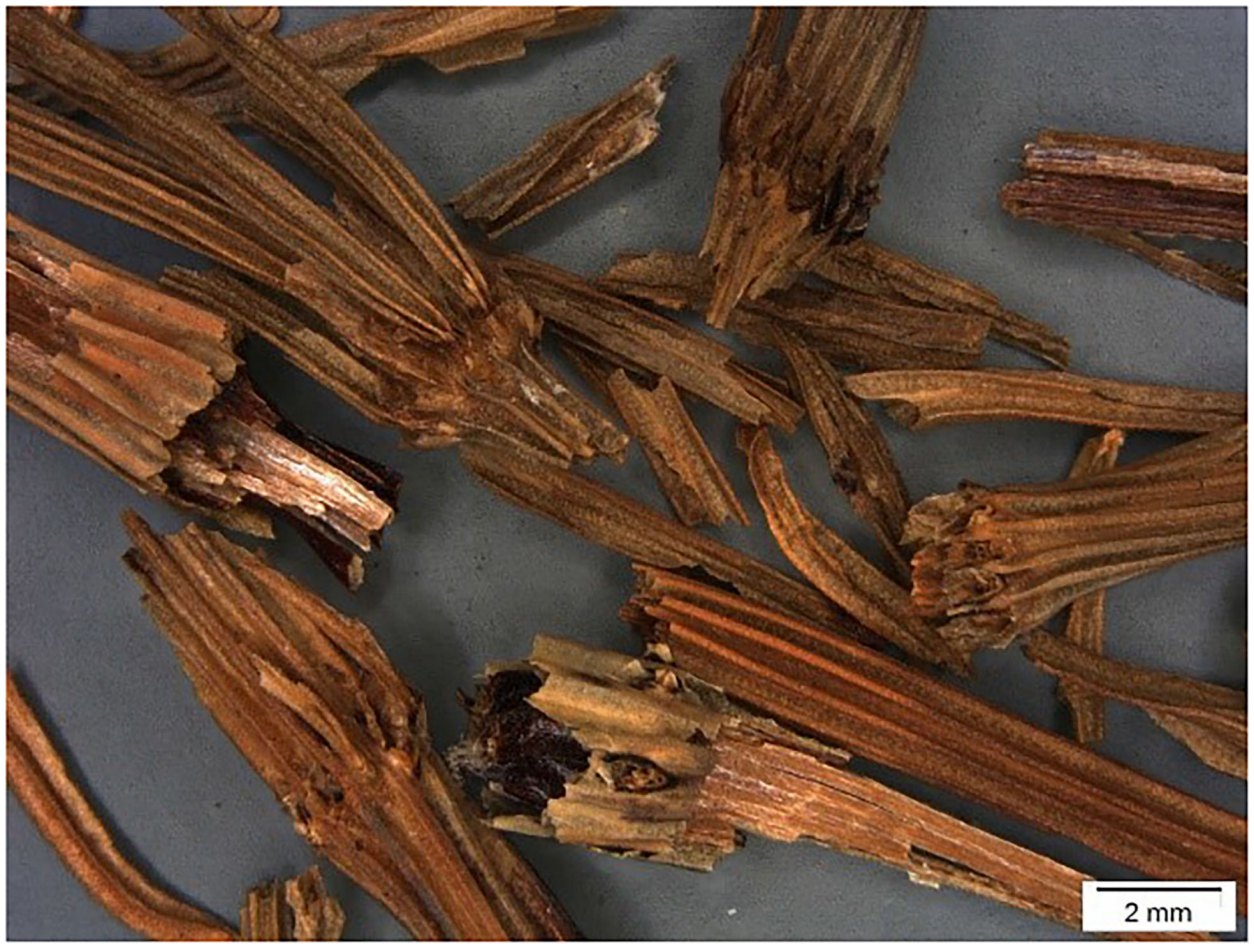
Light microscope picture of parts from common horsetail (*Equisetum arvense*).

The light microscope picture of untreated common horsetail (*E. arvense*) is given in Figure 1.

#### SEM Microscopy

**Figure 2:**
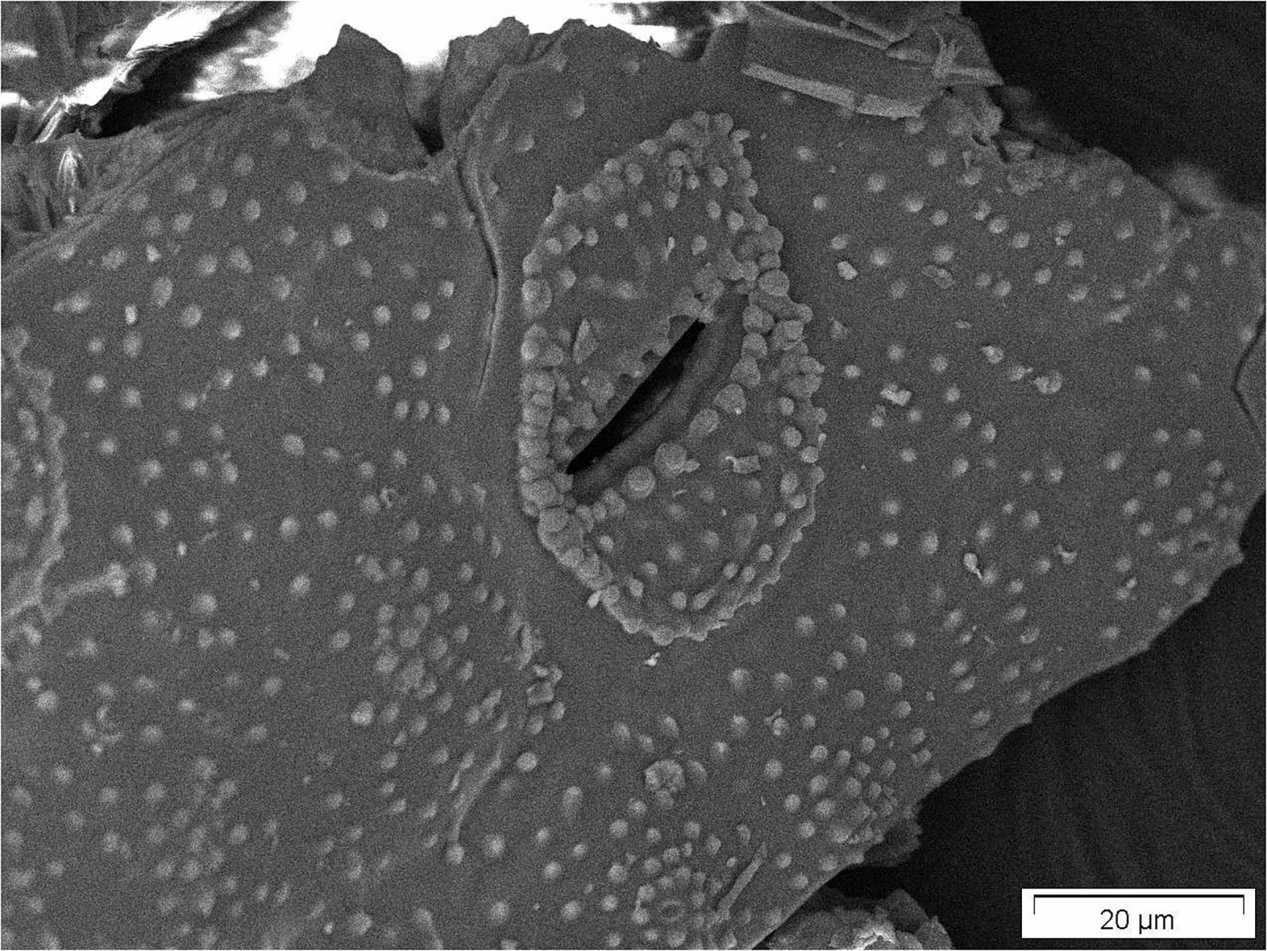
Stomata of just dried common horsetail showing nicely subcuticular incorporated silica particles.

Horsetail SEM pictures, which have just been dried, reveal the full biological structures *(just dried)* and show, e.g., stomata with clearly shown subcuticular incorporated silica particles (Figure 2).

**Figure 3:**
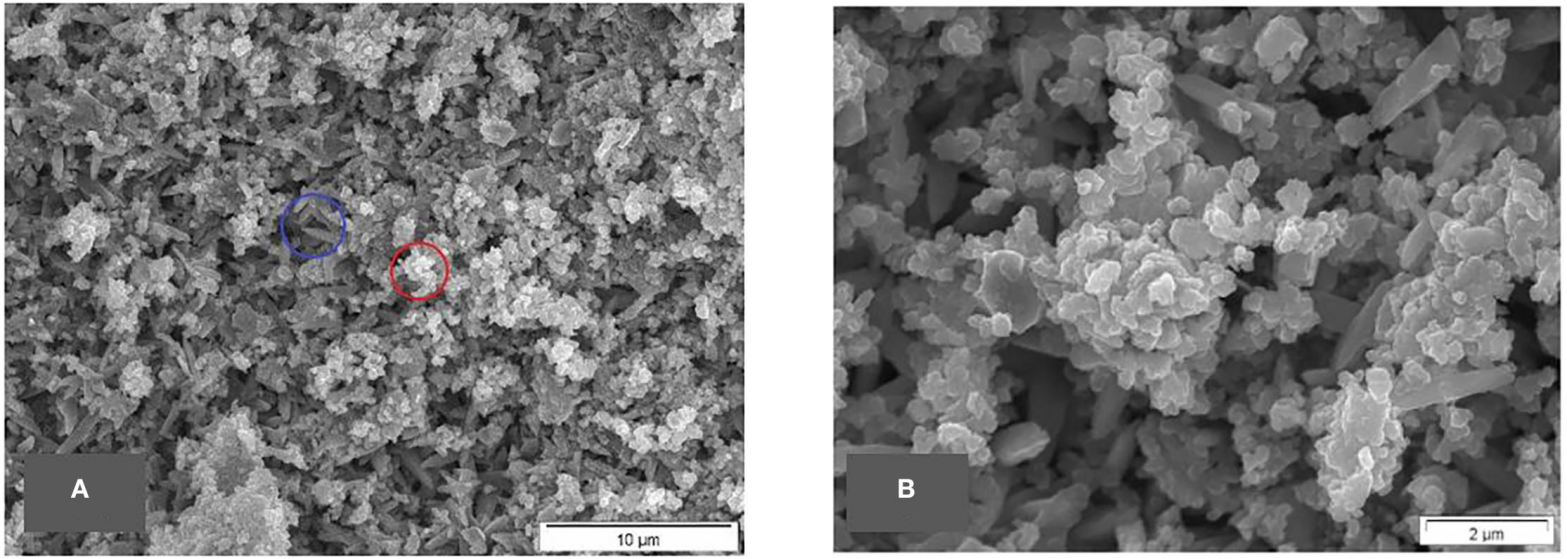
**(A,B)** SEM micrographs of calcined raw materials/washed common horsetail, typical silica structure highlighted by a red circle, structures from other material highlighted by a blue circle.

Following calcination and washing and with greater magnification, the structure of inorganic residues was examined. These pictures (Figures 3A,B) show that besides the structures which are typical for amorphous silica, there is also some crystalline matter.

**Figure 4:**
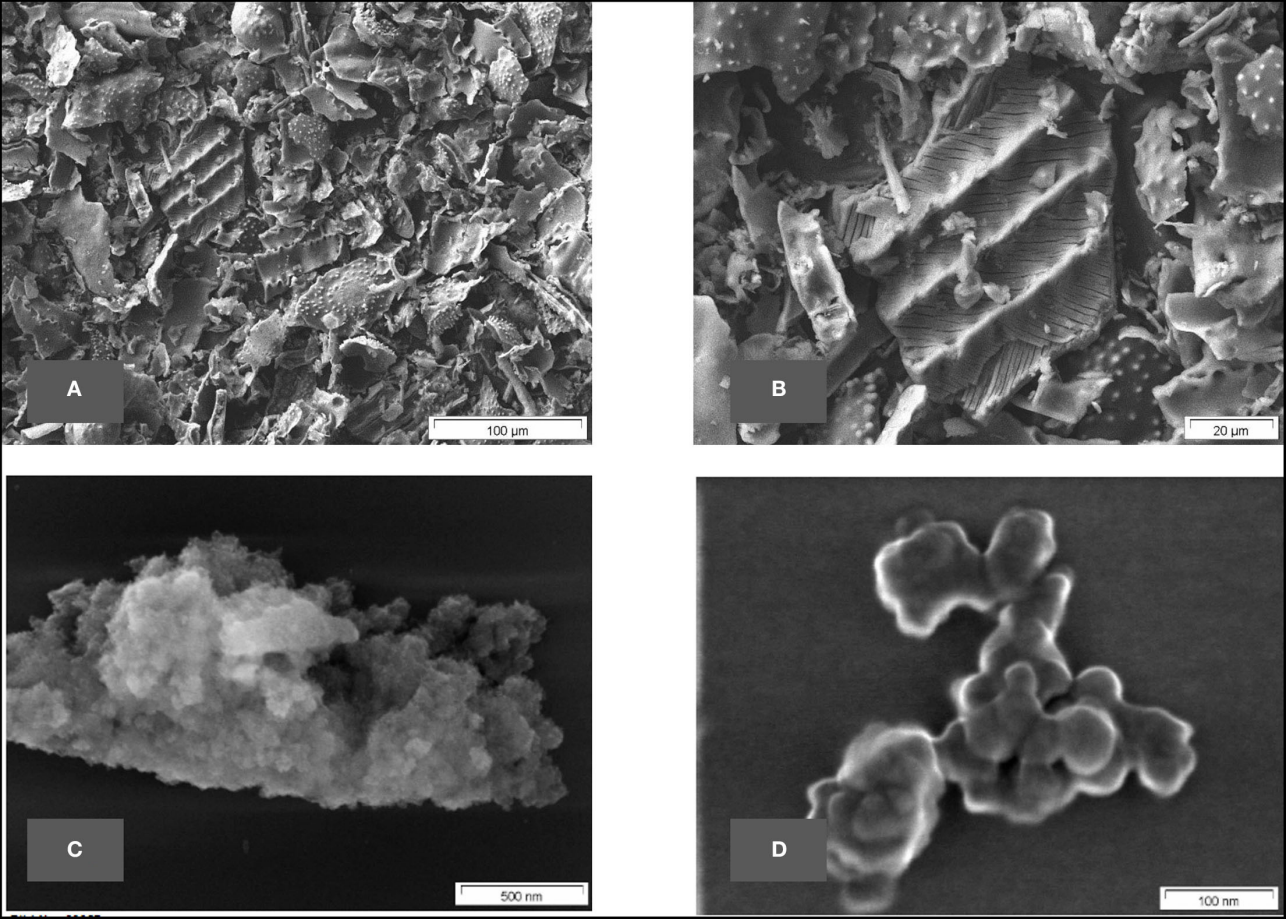
**(A–D)** SEM micrographs of final sample of common horsetail.

At the end of the full sample preparation protocol as described above (final sample), biological structures still can be seen in the lower magnifications but, on greater magnification, the typical structure of amorphous silica can be identified. A series of SEM micrographs at different magnifications is depicted in Figures 4A–D.

#### BET Surface Area

The surface area of the final sample was evaluated according to the BET method, the value of 146 m^2^/g (multipoint) for this horsetail-derived BAS is typical for nanostructured amorphous silica. This measurement corresponds very well with the SEM and TEM pictures, which also reveal a nanostructured biogenic amorphous silica (BAS).

#### SEM-EDX Analysis of the Calcined Raw Material

The SEM-EDX-analysis of the calcined raw material horsetail revealed, besides silicon and oxygen, the presence of Mg, P, S, Cl, K, and a large amount of Ca. This is consistent with the elemental analysis of the raw material described above.

The SEM-EDX-analysis of the samples, which had been prepared according to the above-described digestion method (i.e., final sample), only shows the presence of silicon and oxygen. Thus, this method of SiO_2_ extraction was shown to be highly effective as well as being rather mild.

#### SEM-EDX—Quantitative Analysis—of the Final Sample of Horsetail

**Figure 5:**
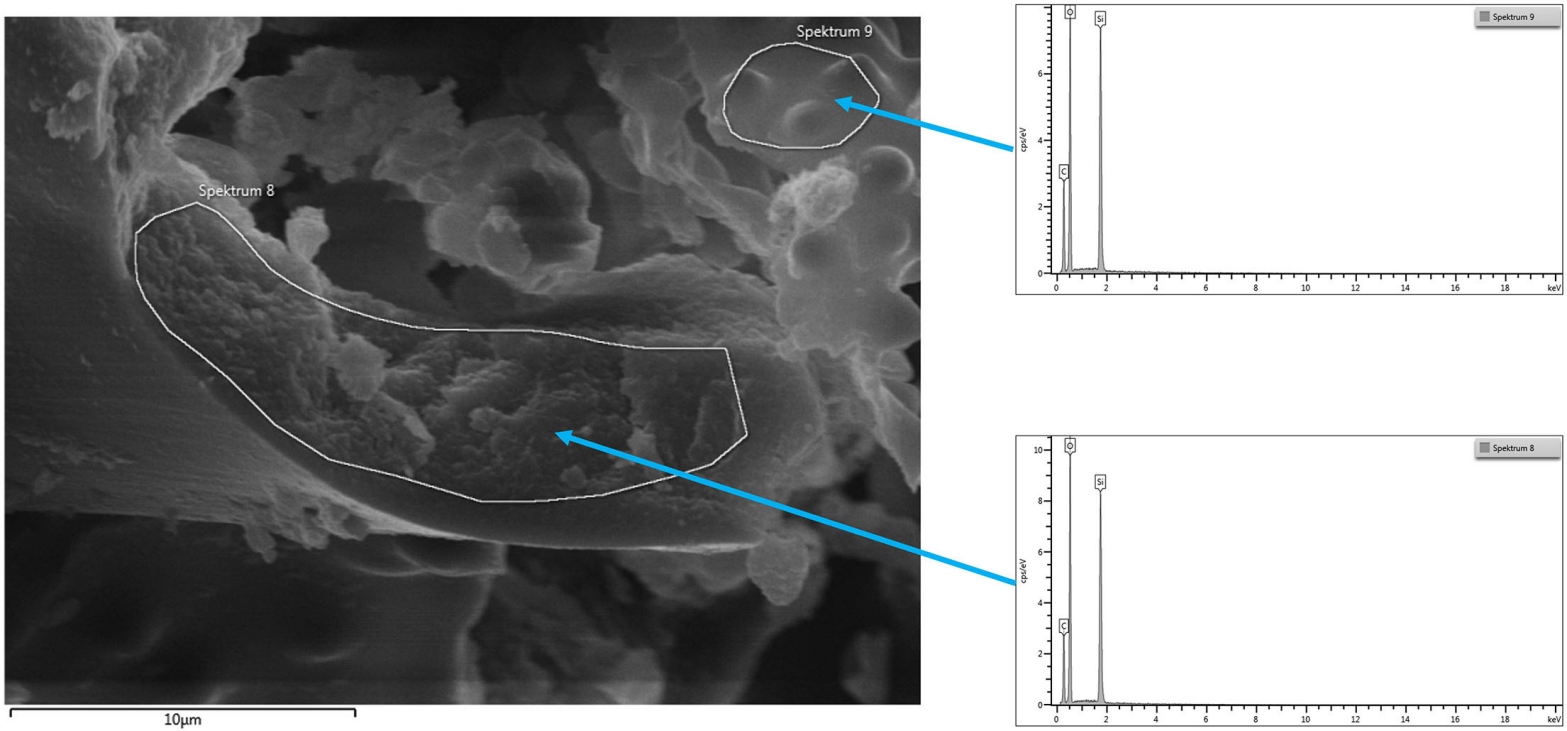
SEM micrograph common horsetail final sample with areas taken for EDX.

The *final sample* shows in the EDX spectrum the presence of Si and O only; thus, confirming the identity as pure silica. Figure 5 shows an SEM micrograph indicating the areas from which the spectra are taken.

#### TEM Microscopy

**Figure 6:**
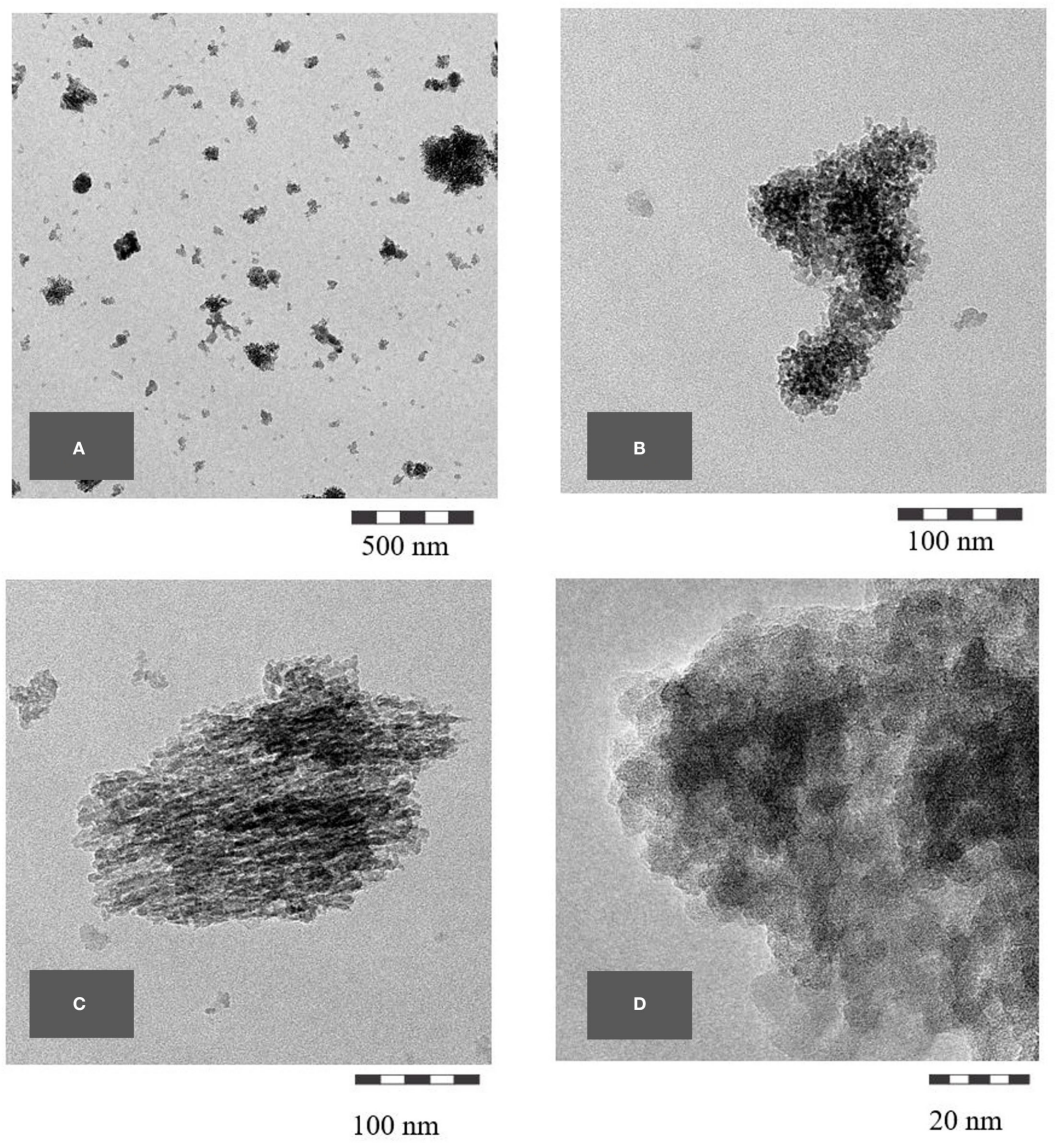
**(A–D)** TEM micrographs of common horsetail final sample.

To visualize the smallest structures within the BAS derived from the horsetail sample, TEM micrographs have been performed as well as the SEM micrographs. The images were prepared only on the *final sample*. The TEM micrographs reveal nanostructured amorphous silica (BAS) consisting of fused primary structures in the range of a few nanometers. Figures 6A–D shows the TEM micrographs at different magnifications.

### Results With Oat Husk

Following the same initial procedure, as was used for the common horsetail, a quantitative analysis of the oat husk raw material was performed in the first stage to get an overview of the composition of the inorganic matter (as shown in Table 1).

For the electron micrographs, the sample preparation was focused on obtaining a pure sample. Again, this was performed similarly to the method described for the sample preparation of the horsetail samples. During this sample preparation, no additional quantitative analysis was performed.

However, the relatively coarse oat husk particles did not appear to readily break down prior to the calcination step, i.e., sedimentation and decantation were very effective without the obvious loss of material. Thus, several samples had been quantitatively analyzed for the loss by calcination with an average value of ~96.1%, i.e., ~4% of the final sample = silica could be recovered (as shown in Table 1). This accords very well with values in the literature where it is reported to be about 5% of silica based on dry oat husk (7, 8).

#### Optical Microscopy of Oat Husks

**Figure 7:**
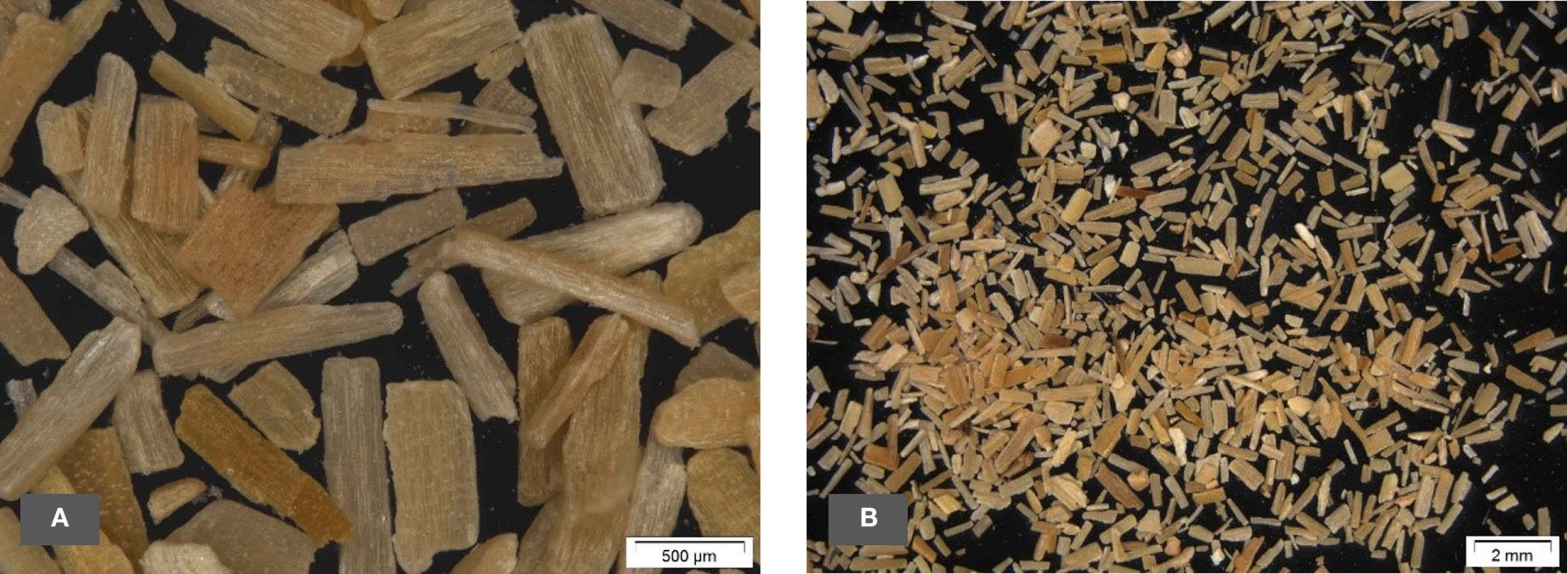
**(A,B)** Light microscope pictures of oat husks.

Light microscope pictures of the untreated oat husks are given in Figures 7A,B.

#### Scanning Electron Microscopy

**Figure 8:**
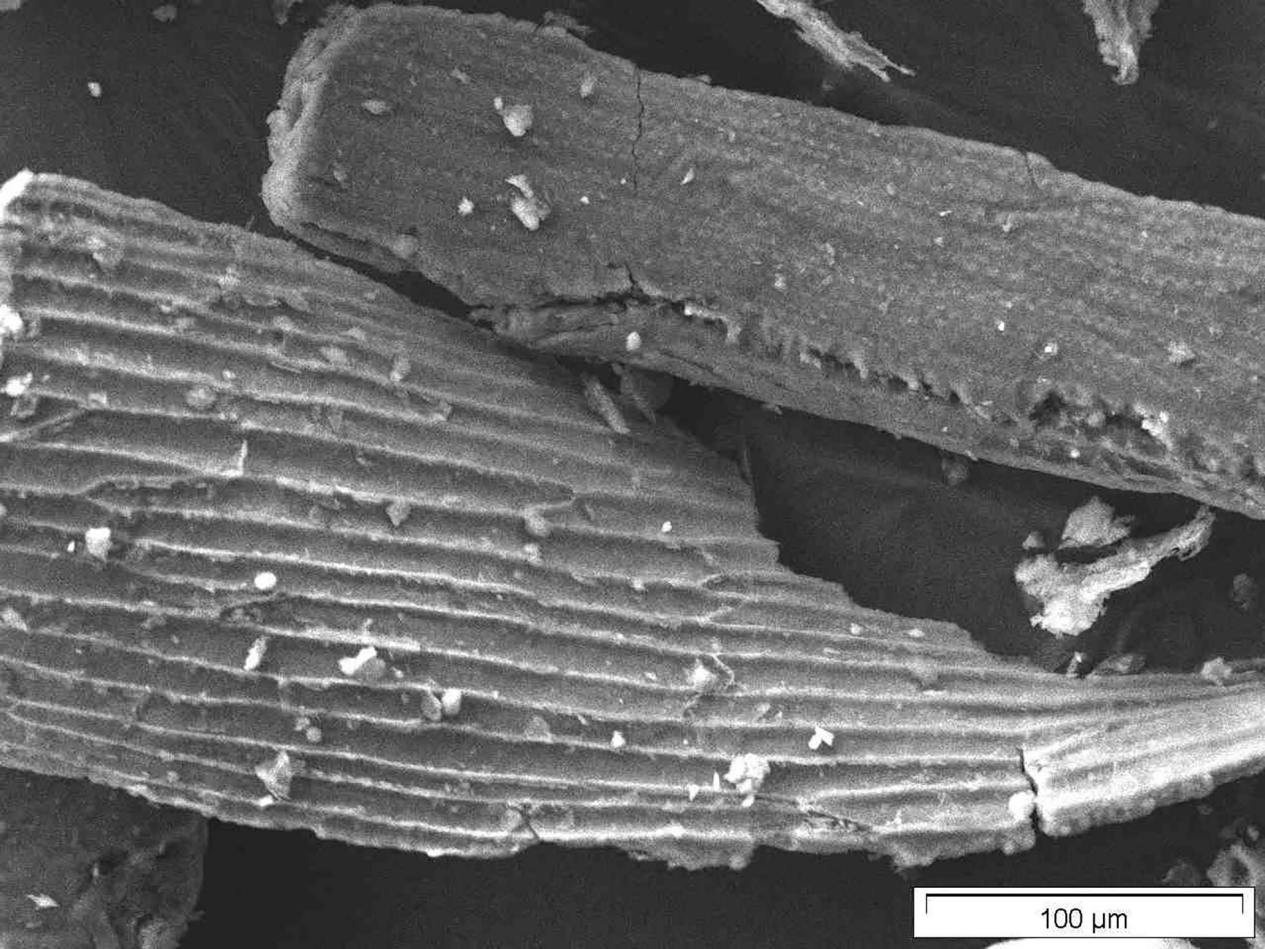
SEM micrograph of out husk (just dried).

**Figure 9:**
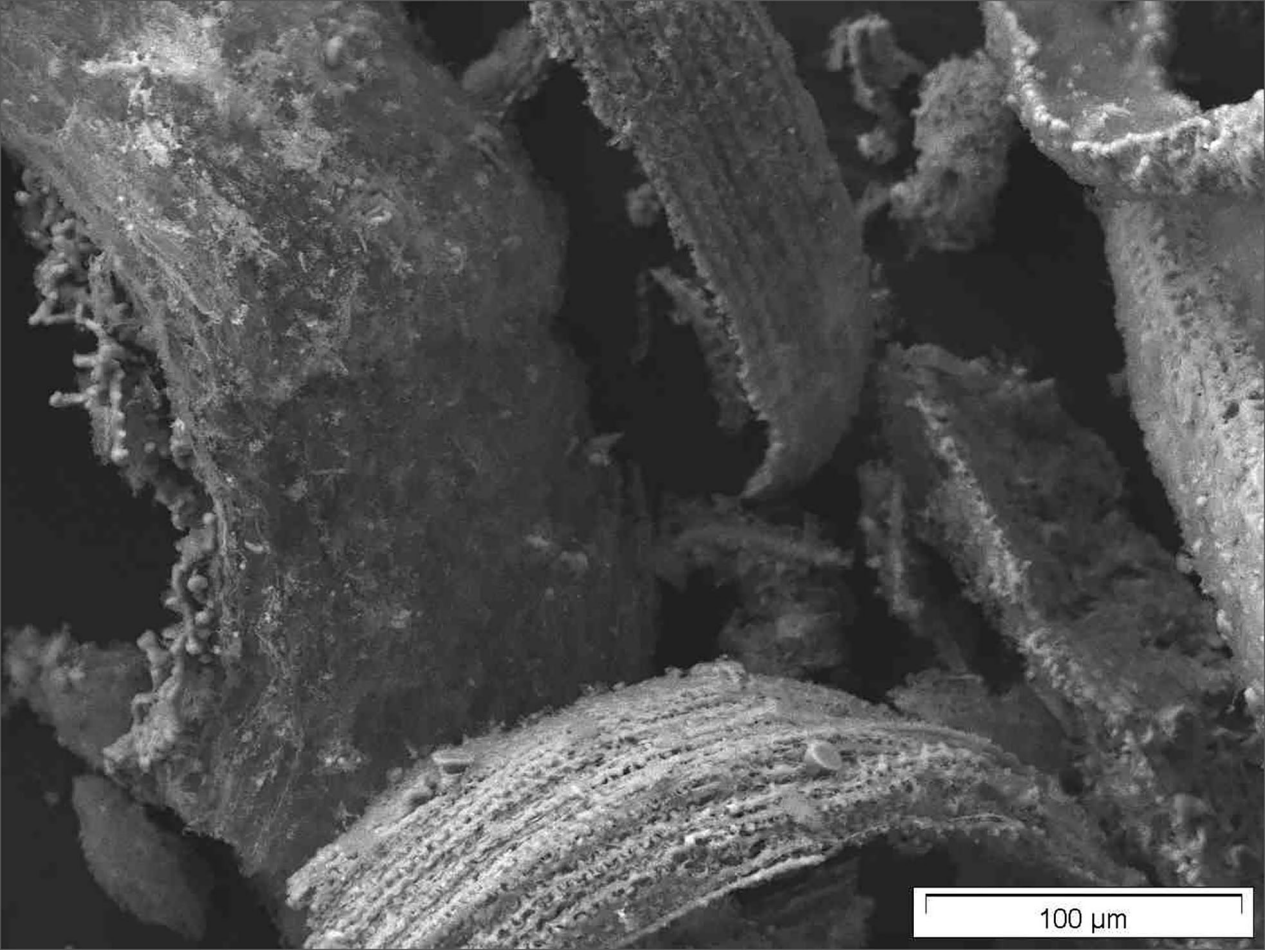
SEM micrograph of calcined oat husk (calcined raw materials).

**Figure 10:**
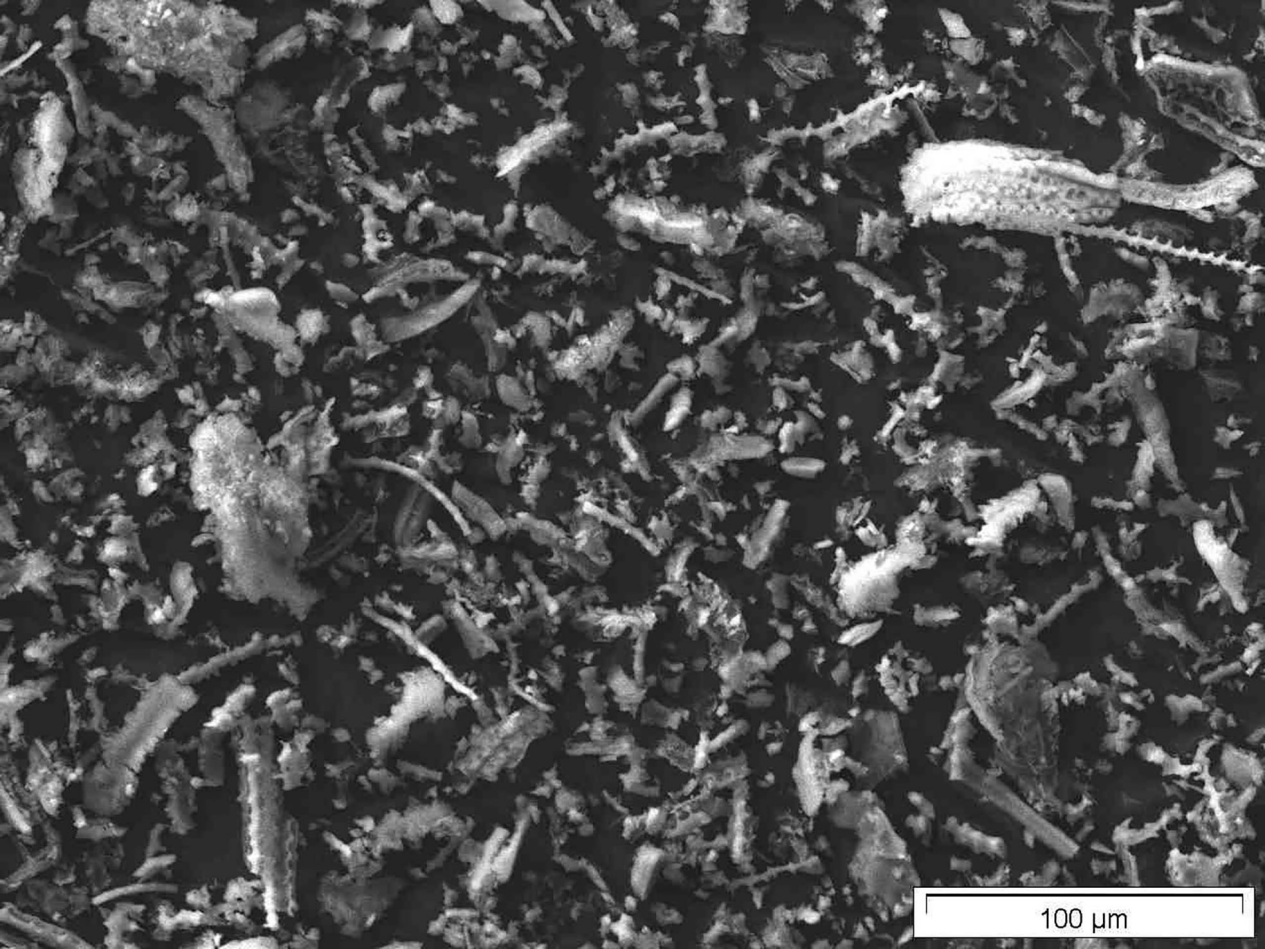
SEM micrograph of final sample of oat husk.

Figures 8–10 show SEM micrographs of oat husks at different stages of sample preparation at low magnification.

**Figure 11:**
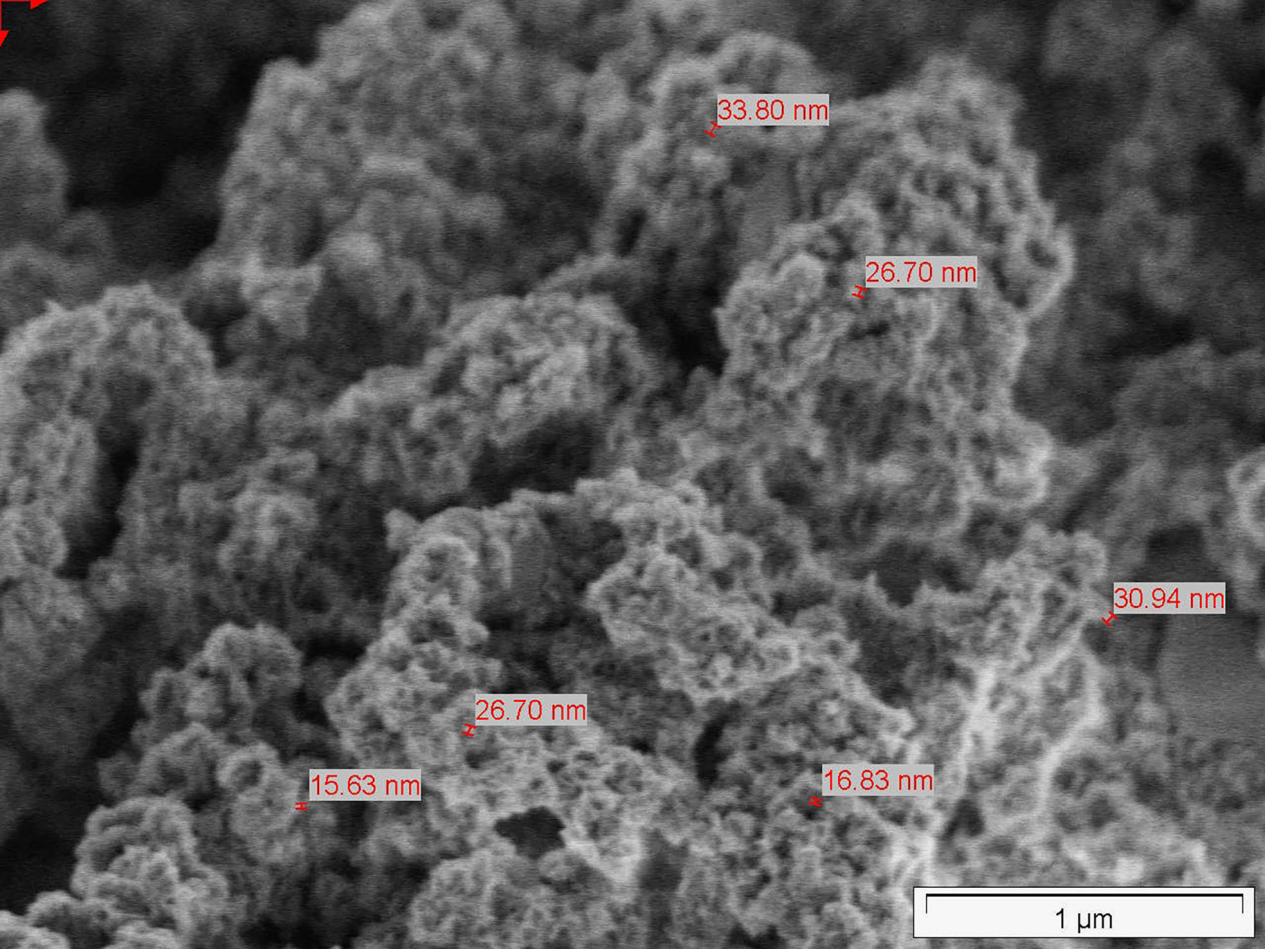
SEM micrographs of calcined oat husk w/o digestion (calcined raw materials/washed) exhibiting nanostructures. Even not as clear as in the final samples they already be identified.

Close examination of the sample of oat husk, which had been only just calcined (540°C) and washed with DI water, revealed easily identifiable nanoscale structures (Figure 11).

**Figure 12:**
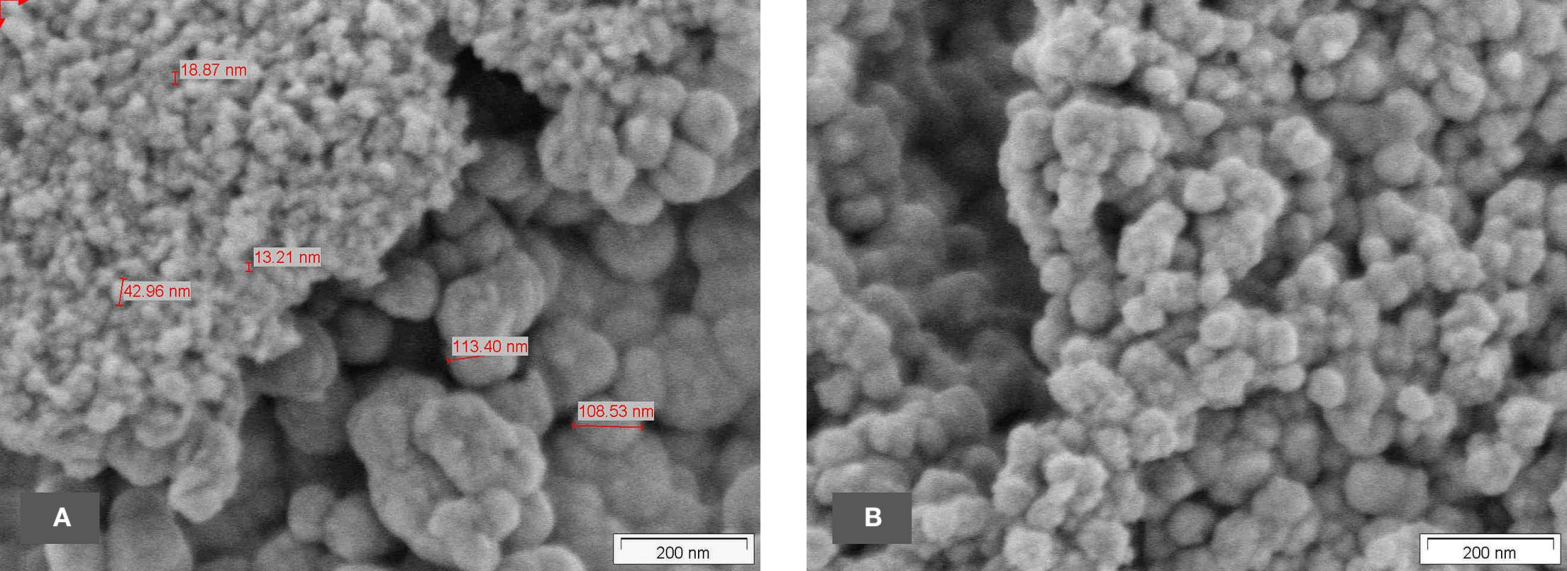
**(A,B)** SEM micrographs of the final sample of oat husk.

When treated with hydrochloric acid, thus transferring the cations into their corresponding readily soluble chloride salts, and after calcinating and thorough washing, the silica nanostructures can be very clearly seen in Figures 12A,B. As previously noted, there are a variety of differently-sized primary structures. The interpretation of the measured BET surface area (multipoint) of ~114 m^2^/g is, therefore, an average value of a range of very small and far larger amorphous silica structures.

In the samples prepared without HCl treatment, nanostructures can already be seen but still seem to be covered by some non-silica material and are difficult to identify clearly. In the fully-treated samples, however, the purified silica reveals unambiguously its nanostructure (e.g., Figures 6, 12).

#### TEM Microscopy

**Figure 13:**
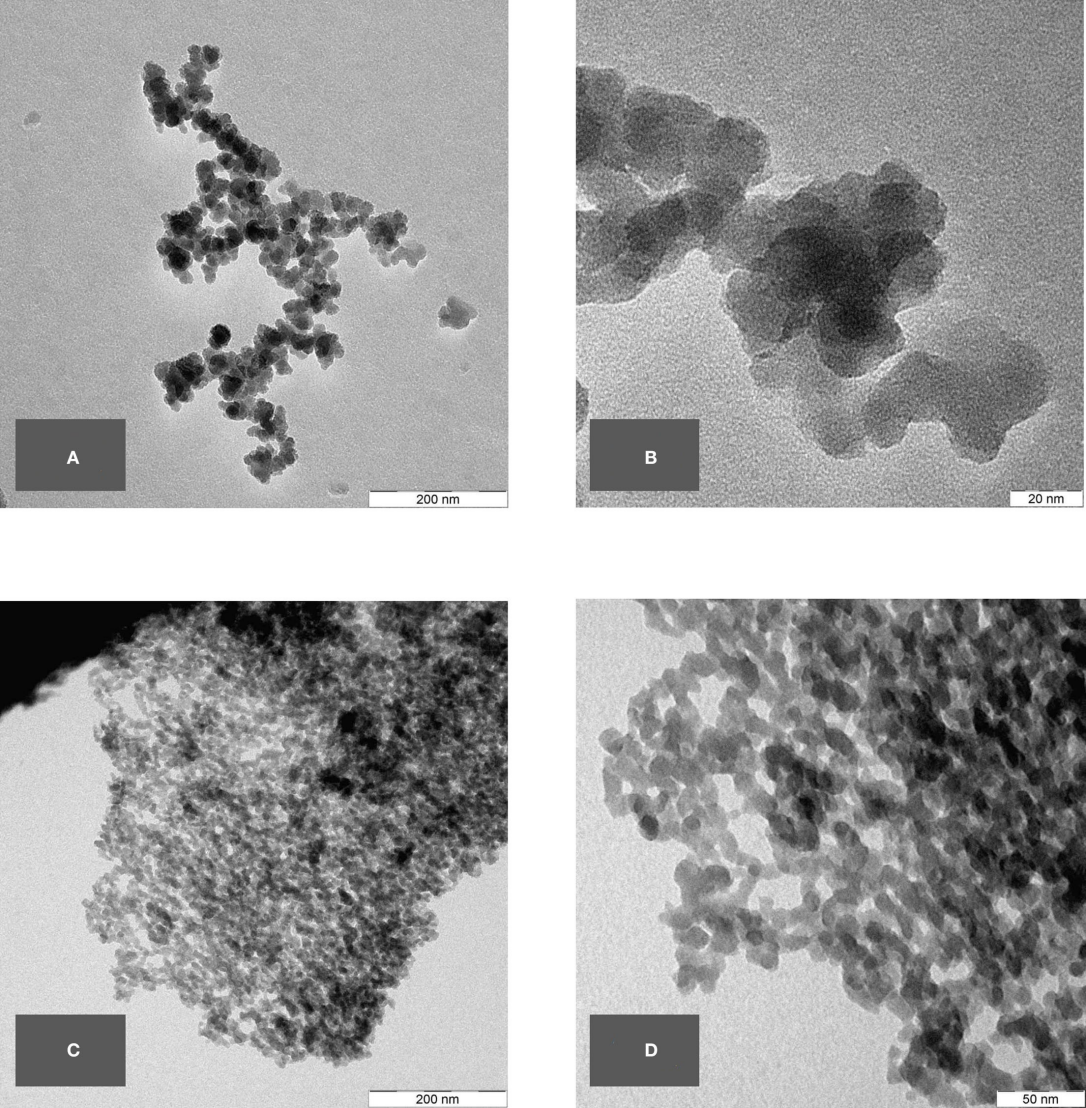
**(A–D)** TEM micrographs of final sample from oat husk at different magnifications.

As with the horsetail, TEM images were prepared in addition to SEM pictures to investigate the structure of oat husk-derived BAS in more detail at higher magnifications. Figures 13A–D show very clearly that the nanostructure of these samples is very similar to the structures of SAS.

Several ATEM-EDX spectra of the final sample revealed that besides silicon and oxygen, only very minor impurities are present, i.e., about 0.06–0.25% (atom) K, up to 0.23% (atom) Al, and up to 0.06% (atom) Ca.

### Comparison of BAS to Commercial SAS Products Using TEM and BET

In terms of the chemical composition of SiO_2_ and its amorphous structure, as well as their nanostructured appearance with similar size ranges in the corresponding primary structures, both BAS and SAS are highly comparable.

TEM pictures, as shown below, demonstrate the similarity between BAS and SAS. Thus, it is not possible to easily distinguish between BAS and SAS based on the results obtained by this described analytical methodology. The specific surface area (BET) also shows that the examined BAS substances are NMs with values >100 m^2^/g.

#### TEM of Commercial SAS Grades

**Figure 14:**
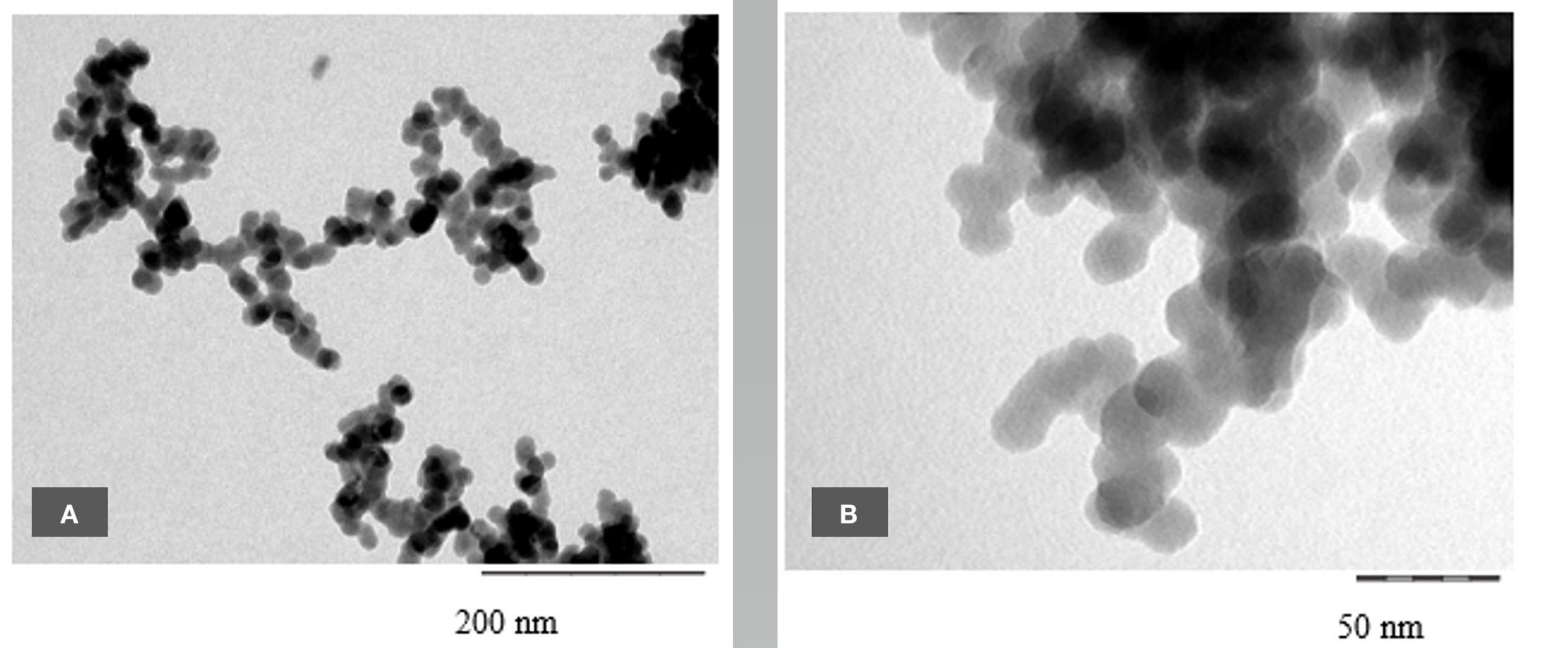
**(A,B)** TEM micrographs of SIPERNAT® 160 at different magnifications.

**Figure 15:**
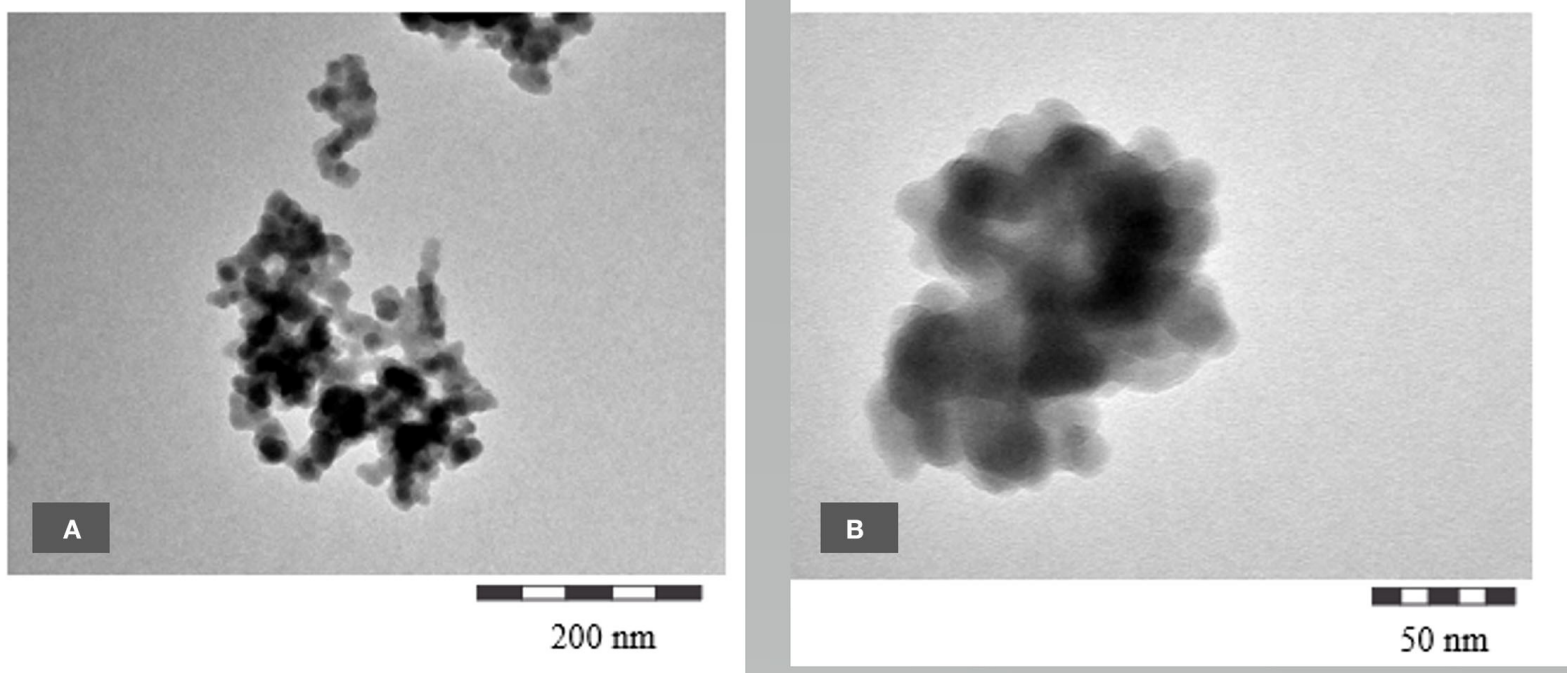
**(A,B)** TEM micrographs of SIPERNAT® 22 at different magnifications.

Figures 14, 15 and Supplementary Figure 1 show TEM micrographs of different commercially available pyrogenic and precipitated SAS grades. The following commercially available SAS grades have been used.

Precipitated SAS: SIPERNAT® 160 BET 170 m^2^/g

Precipitated SAS: SIPERNAT® 22 BET 180 m^2^/g

Fumed SAS: AEROSIL® 200 BET 200 m^2^/g

[Characteristic BET-surface data taken from (9, 10)]

The images show clearly a very similar nanostructure compared with the BAS grades described above.

## Discussion

### Background and Literature Review

As early as 100 years ago, silica was found to occur naturally in the human body (3). Based on this observation, it can be assumed that this form of silica has been routinely taken up by the daily diet (11). The European Food Safety Agency (EFSA) notes the Si content in their Scientific Opinion of the Panel on Food Additives and Nutrient Sources added to Food from 2009 (12) for several cereals, e.g., oat 3,910–4,310 mg/kg, barley 2,610–2,720 mg/kg, wheat flour 2,610–2,720 mg/kg, and for polished rice 55–57 mg/kg dry weight.

Martin (13) found that the human body contains about 1.5 g of SiO_2_, presumably both in the particulate and dissolved forms (this is in accordance with Merck, 1921). Robberecht (14) provided a comprehensive overview of silicon in food (meat, see food, cereals, milk, vegetables/fruit, and drinks), its content, and bioavailability. The fate and biological responses to SAS in food have been recently described by Yu (15) and an advanced intestinal *in vitro* model has been addressed by Hempt (16). Due to the uptake of silica from different sources of biogenic and artificially generated origin, it is almost impossible to differentiate between the two sources. Theoretically, all sources should have different ratios of Si/O-isotopes; thus, a distinct separation might be possible but to date, this has only been done within a rather limited narrow field of observations (17).

It needs to be noted that in the literature, one needs to be cautious as the word “silica” has been applied to both crystalline (quartz) and amorphous species (1, 18) and sometimes without further clarification. In this article, we have been careful to discuss only amorphous particulate silica.

As noted earlier, amorphous silica can be found in the skeletons of algae (diatoms) (19–26), even in deep-sea sponges and molluscs (27).

Interestingly, there is a DFG Research Unit 2038 “Nanomee” (http://www.nanomee.de/) in Germany that is tasked “to obtain a detailed understanding of the biomolecule-controlled nano- and microscale processes that enable diatoms to biosynthesize their species-specifically patterned SiO_2_-based cell walls.” (28–31).

Plants require BAS—*via* biomineralization—for their growth and strength in so-called biogenic silica structures (32–40).

So-called phytogenic silicas are characteristic of different plant species and of the different functions/locations where they occur.

Amorphous silica is essential for plant growth in terms of not just contributing their physical strength to assist plant structure, but also for their health. Surprisingly, the most abundant and ubiquitously-found silica source in nature, quartz, however, is not available to plants due to its very low water solubility. Therefore, plants have to rely on the presence and adequate concentration of amorphous silica in the soil. The grass types—among them wheat, rye, oat, rice, and others—grow smaller and are more susceptible to diseases when the accessible silica has been substantially reduced by extensive farming. BAS has been found to enhance the resistance of plants to biotic stress, e.g., diseases, such as powdery mildew and pests, such as stem boring insects (41, 42).

Additionally, it has been found that amorphous silica is important for the water storage capacity of the soil and its ability to release immobilized phosphorous, thus making it available to plants (43–50).

The common horsetail is considered to contain the highest percentage of amorphous silica of all living plants, up to 25% (34)—with especially high values in the stabilizing joints. It is remarkable to note that its evolutionary ancestors, from ~300 million years ago, reached up to well above 20 m in height, with almost the same appearance as seen now. This has been only possible due to the biogenic silica structures stabilizing such constructions.

The first TEM images of the BAS in common horsetail were obtained by Perry and Fraser in 1991 (6) and display distinctive structures of different plant areas with certain “tasks” (e.g., leaves and joints). In 2007 (32), TEM images were obtained showing even more clearly the similarity of BAS to SAS in the case of horsetail. Indeed, the TEM images obtained in this study revealed that BAS looks rather like mirror images of SAS.

As for the BAS in grasses, and those in all grains, amorphous silica NMs in rice husk (from *Oryza sativa*) have been the most extensively studied and the subject of many publications (51–53). In the oat plant, the BAS is also mainly concentrated in the oat husk, too. Moreover, oatmeal is considered a viable daily source of silica in modern breakfast. Therefore, for this reason, the oat husk was chosen in this current investigation as the second natural organic source for the determination of BAS. Interestingly, using SEM imaging, it is possible to identify nanostructured silica even in the oat husk ash directly.

The ubiquitous occurrence of BAS and its having a very similar nanostructure to SAS is an interesting aspect that needs to be considered and should be taken into account in any discussions about the safety of NMs, especially in the context of the oral uptake of SAS in food, feed, and cosmetic applications.

Taken all together, commercial SAS forms are not entirely new NMs with unknown properties, but are well-studied materials that have been in use for decades without changes in their basic physicochemical structure.

### Interpretation of the Presented New Studies With Common Horsetail and Oat Husk

In this current investigative series performed on oat husk and common horsetail, we have examined the nanostructure of BAS by SEM and TEM analysis and compared them with the nanostructure of commercially available SAS materials. Furthermore, we have also performed BET analysis in both types of natural and SAS products.

Scanning electron microscopy is an established method to evaluate the structure of materials down to the nanoscale. In our SEM overview images, it could be shown that the structural analogy between SAS and BAS can be found throughout the whole of the biological sample and not only in isolated locations. Using TEM images, which are suitable to show the structure at even greater magnifications, a direct comparison between BAS and SAS was possible and demonstrated that BAS is structurally very similar to SAS. Thus, the naturally-occurring BAS may even be considered a bio-analog to SAS. This is also confirmed by a comparison of the surface area of both these two amorphous silica types.

Based on our findings and the identical chemical composition, there seems no reason to differentiate between naturally-occurring BAS, which is present in many food sources and is perceived beneficial to health, and SAS.

## Data Availability Statement

The original contributions presented in the study are included in the article/Supplementary Material, further inquiries can be directed to the corresponding author/s.

## Author Contributions

All authors listed have made a substantial, direct, and intellectual contribution to the work and approved it for publication.

## Conflict of Interest

GL, C-PD, KS, TS, and NK were employed by Evonik Operations GmbH.

## Publisher's Note

All claims expressed in this article are solely those of the authors and do not necessarily represent those of their affiliated organizations, or those of the publisher, the editors and the reviewers. Any product that may be evaluated in this article, or claim that may be made by its manufacturer, is not guaranteed or endorsed by the publisher.

## Footnotes

^1^Definition according to ISO/TS 80004-1.

^2^Alternatively, the abbreviation bASi is also used in soil sciences.

## Supplementary Material

Supplementary Figure 1 **(A,B)** TEM micrographs of AEROSIL® 200 at different magnifications.
