# Supplementary figures and images for: Comparison of Biogenic Amorphous Silicas Found in Common Horsetail and Oat Husk With Synthetic Amorphous Silicas
Source: Front Public Health. 2022 Jun 22;10:909196. doi: 10.3389/fpubh.2022.909196 (PMC9257020; doi:10.3389/fpubh.2022.909196)

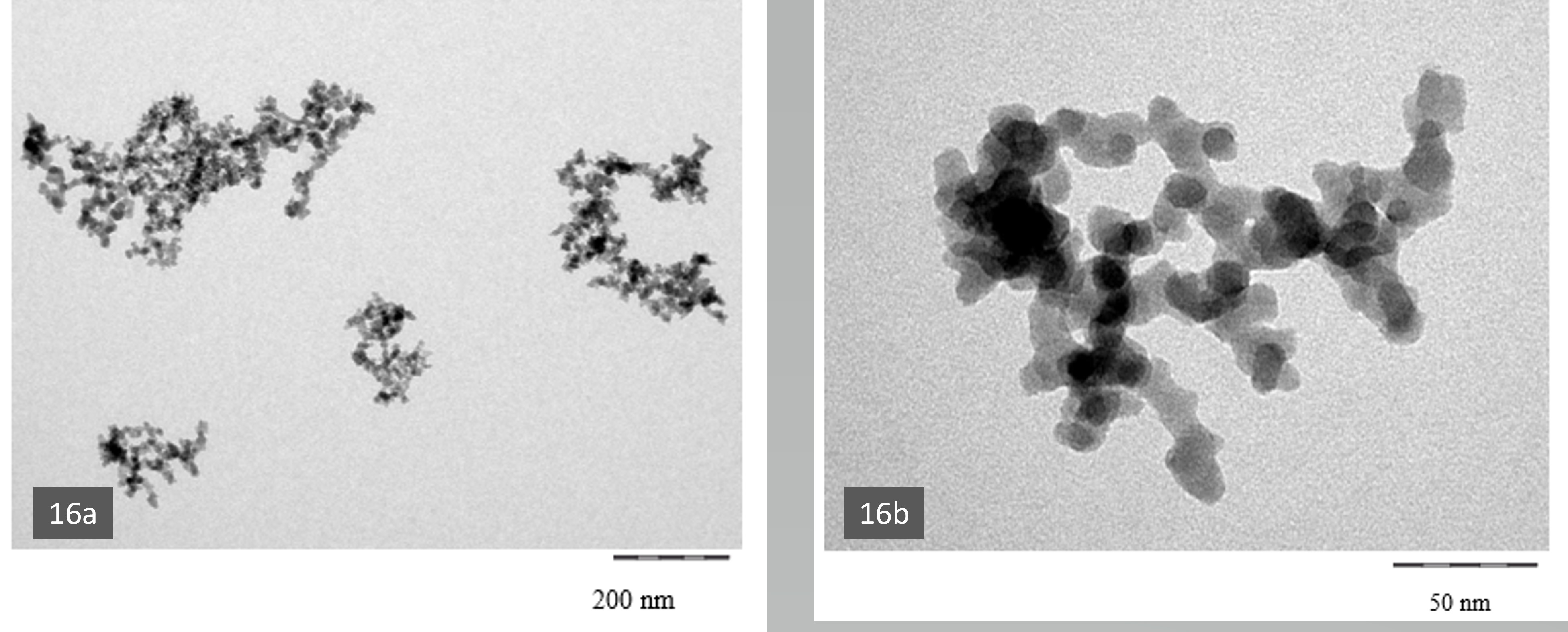

Supplement: Supplementary Figure 1 — (A,B) TEM micrographs of AEROSIL® 200 at different magnifications. [file Image_1.JPEG]
